# The Experience of Melanoma Follow-Up Care: An Online Survey of Patients in Australia

**DOI:** 10.1155/2014/429149

**Published:** 2014-11-19

**Authors:** Janine Mitchell, Peta Callaghan, Jackie Street, Susan Neuhaus, Taryn Bessen

**Affiliations:** ^1^School of Population Health, Level 11 Terrace Towers, 178 North Terrace, The University of Adelaide, P.O. Box DX 650 205, Adelaide, SA 5005, Australia; ^2^Royal Adelaide Hospital, North Terrace, Adelaide, SA 5000, Australia

## Abstract

Investigating patients' reports on the quality and consistency of melanoma follow-up care in Australia would assist in evaluating if this care is effective and meeting patients' needs. The objective of this study was to obtain and explore the patients' account of the technical and interpersonal aspects of melanoma follow-up care received. An online survey was conducted to acquire details of patients' experience. Participants were patients treated in Australia for primary melanoma. Qualitative and quantitative data about patient perceptions of the nature and quality of their follow-up care were collected, including provision of melanoma specific information, psychosocial support, and imaging tests received. Inconsistencies were reported in the provision and quality of care received. Patient satisfaction was generally low and provision of reassurance from health professionals was construed as an essential element of quality of care. “Gaps” in follow-up care for melanoma patients were identified, particularly provision of adequate psychosocial support and patient education. Focus on strategies for greater consistency in the provision of support, information, and investigations received, may generate a cost dividend which could be reinvested in preventive and supportive care and benefit patient well-being.

## 1. Introduction

Globally, Australia has the highest incidence of melanoma, with annual rates continuing to rise [1]. Individuals with a primary melanoma have 8–12% risk of developing a second primary melanoma and an increased risk of developing a nonmelanoma skin cancer [2–5], and therefore posttreatment monitoring for recurrence and new primary melanomas is important. The purpose of follow-up is to detect recurrence and/or progression at an early treatable stage, identify treatment-related morbidity (e.g., lymphoedema), identify new melanoma or nonmelanoma skin cancers, and provide reassurance and education [6]. Good practice in follow-up includes effective coordination of care, consistency in care provision [7, 8], evidence-based testing, and psychosocial support [6–9]. Patient perceptions can provide valuable insight into the quality of melanoma follow-up care and identify potential areas for improvement.

Quality of patient care can be defined in both technical and interpersonal terms [10]. Here, “technical” refers to best practice based on current evidence coupled with care providers' knowledge, judgment, and skill in implementation [10]. The 2008 Australian Cancer Network Melanoma Guidelines publication describes best practice guidelines for melanoma follow-up, including judicious use of imaging and blood tests, patient education for detection of recurrence and new primary melanomas, and scheduling follow-up visits with health professionals, on the basis of stage of the cancer, familial history, and patient ability to perform self-examination [6].

The success of technical care depends, in part, on the management of interpersonal care [10], including factors such as communication, empathy, and trust. The subjective nature of interpersonal care makes it a difficult construct to measure; therefore, “best practice guidelines” in this area can be difficult to formulate and implement [10]. Patient satisfaction can be one measure, with self-reported measures of satisfaction helping to determine the values patients themselves associate with quality of care [11]. Measures of patient satisfaction consist of both cognitive evaluation and emotional response to the structure and process and outcome of health services received [12]. The amount and clarity of information received correspond with the cognitive evaluation, whilst the emotional responses encompass psychosocial factors such as receiving adequate support. The psychosocial interventions advocated by the 2008 Australia and New Zealand clinical practice guidelines include cognitive-behavioural group therapy and psychoeducation and access to support groups.

Previous research on melanoma patients' experiences in Australia at follow-up consults recommended a tailored approach to follow-up care with adequate provision of information enabling patients to participate in shared decision-making [9]. Overall high levels of satisfaction with follow-up care were reported by Morton et al. [9]; however, the sample was restricted to patients adhering to follow-up schedules in specialist melanoma centres, potentially targeting only those patients who had a positive experience. Similarly, a systematic review of 15 studies examining the psychosocial aspects of melanoma follow-up care reported high levels of patient satisfaction although none of the studies were Australian [13]. Carter et al. argue that effective delivery of follow-up in Australia and elsewhere is challenging [14]. In particular, the guidelines are based on low-level evidence leading to variation in practice. This study was conducted to investigate if new knowledge in follow-up care in Australia was essential towards fulfilling the health needs of melanoma patients.

We used an online survey distributed across a variety of settings, including nonmetropolitan and multistate, for participant recruitment. Our study aimed to collect the views of patients with a broad base of experience in melanoma follow-up care in Australia, investigating patient perceptions of both the technical and interpersonal aspects of the quality of their follow-up care.

## 2. Materials and Methods

### 2.1. Participants

Purposive sampling ensured that only patients with experience of melanoma follow-up care participated. Participants were patients treated in Australia for a primary melanoma since 1 January 2007. Selection criteria excluded patients diagnosed in the previous year, as such patients were deemed to be in “treatment phase” and not in follow-up care.

One hundred and fifty patients accessed the questionnaire with 33 incomplete surveys and 53 surveys that were excluded as the participants were diagnosed before 2007. The remaining 64 participants were included ensuring that only current data on follow-up care was used for this analysis. Patient characteristics are listed in Table 1.

### 2.2. Materials

Responses were collected using a web-survey through SurveyMonkey. An online information sheet preceded the questionnaire to inform participants about the study and obtain consent. Anonymity was assured and no payment was offered. The 40-question survey, predominantly with “tick box” format, collected information about patient demographics, nature of the melanoma, treatment details, and nature of follow-up care, including the Assessment of Survivor Concerns, as developed by Gotay and Pagano [15]. A pilot study with 5 melanoma patients recruited through a surgical oncologist tested the survey before dissemination. Pilot study participants emphasised the emotional aspects of melanoma follow-up care. As a result, additional questions to assess this factor were included. Free-boxes permitted participants the opportunity to elaborate on their survey responses and contribute further comments. These open-ended comments comprise the study's qualitative data.

### 2.3. Procedure

Australian state and national organisations involved in melanoma care were approached to support the project ensuring a range of patient experiences, unrestricted by a single specialist centre. The following organisations supported advertisement and recruitment through their websites and networks: Melanoma Patients Australia, the Cancer Council of South Australia and the Cancer Council of Australian Capital Territory, the Australia and New Zealand Melanoma Trials Group, the Melanoma Institute Australia, the National Melanoma Symposium (Melbourne, 2012), Melanoma WA, and Sunbedban. An additional media release in a major South Australian newspaper was also conducted.

Ethics approval was obtained from the University of Adelaide Human Research Ethics Committee (number H-2012-037). Data collection occurred for the period of June-September, 2012. Analysis was supported by IBM SPSS version 19, Armonk, NY, USA (quantitative analysis) and NVivo 10.1 (qualitative analysis) [16].

### 2.4. Qualitative Analysis

In thematic analysis, as detailed by Braun and Clarke [17], the data were independently coded by two authors (Peta Callaghan and Jackie Street) with iterative discussion to determine concurrence and resolve discrepancies. After open coding, overarching themes were derived, condensing smaller categories: for example, categories relating to mental health and emotional issues, including counselling, fear, stress, and need for reassurance were grouped to form the overarching theme and psychosocial factors.

We were interested in exploring patients' experiences of their care, including information needs, degree of anxiety, experience of follow-up care, and perspectives on potential benefit of a follow-up care coordinator. Our analysis therefore focused on patient experience of melanoma follow-up care.

## 3. Results

### 3.1. Patient Satisfaction

Patient satisfaction was determined by three questions that assessed patient experiences of adequate support during follow-up care, patient satisfaction with the amount of information received, and patient preferences for a coordinator to manage follow-up care. Many participants reported that they did not receive adequate support during follow-up (51.6%), that they would have liked to receive more information from their health professionals (64.1%), and that they would like a coordinator to organise their melanoma follow-up care (62.5%).

Whilst the categorical nature of our data restricted multivariate analysis, general response patterns were identified. Participants not supporting the use of follow-up care coordinators also generally reported receiving adequate support and being satisfied with information provision from their health professionals. This trend was also found in the opposite direction: participants supporting the use of a coordinator generally described inadequate support and information provision during follow-up care.

### 3.2. Technical Aspects of Quality of Care

#### 3.2.1. Health Professionals Seen and Frequency of Visits

Surgeons were the most frequently reported health professional seen (32 participants), followed by dermatologists (30 participants), although 8 patients saw both. Intervals between visits varied considerably across the patient group; for example, patients with <1 mm melanomas reported being seen 3 monthly. Although 31 patients reported that they regularly saw a general practitioner, it is possible that the reason for these visits may not have specifically related to melanoma follow-up. A minority of participants (22%) reported regular consults with three or more melanoma specialists.

#### 3.2.2. Diagnostic Tests

In follow-up care (after the initial 12-month treatment phase), the most common diagnostic test described was a computed tomography (CT) scan (41 participants), followed by positron emission tomography (PET) scans (17 participants) and chest X-rays (CXR) (11 participants). Several patients indicated a preference to have more scans during follow-up care. Patients constructed the provision of more scans as a means of reassurance (Box 1). Patient awareness of the superiority of self-examination to monitor future risk was not reflected strongly in our data with only one participant explicitly reporting conducting their own skin checks. Comments from many participants indicated that they were unaware of the benefits of skin checks or how to perform adequate self-examination.

### 3.3. Interpersonal Aspects of Quality of Care

In line with previous research, the most common type of dissatisfaction expressed was with the type of communication and information offered [11]. Sixty-four percent of participants reported they would have liked more information about melanoma, and comments about the quantity and quality of communication dominated the qualitative data.

#### 3.3.1. Information Received

For many participants (40.6%) websites were the main sources of information on melanoma, with the second main source being surgeons (25%). Nearly half of the participants (46.9%) reported not receiving written information, and around 70% reported receiving no information on cancer services, patient support websites, or generic sun protection measures (e.g., avoidance of solariums, use of sunscreens). Thirty-eight participants (59.4%) received a copy of their melanoma pathology report, but only 3 (4.7%) received a written follow-up plan. Box 1 provides examples of participants' comments about information provision. Lack of information was the most commonly reported theme in this category; however, conflicting information and the need for ongoing information due to “forgetting” were also reported.

#### 3.3.2. Communication

Communication was constructed in 2 primary ways: communication between professionals and communication with professionals. Within these categories, three themes were expressed: poor communication, ideal communication, and good communication with poor communication reported as the most common experience (Box 1). Many participants described the type of communication they felt* should* happen. Ideal communication was frequently constructed in terms of “reassurance” for patients. The lack of information (received or perceived) meant many participants accessed information themselves via the Internet; however, participants also indicated that poor communication with their health care professional meant they often did not know what to look for or which information applied to them.

#### 3.3.3. Changed Professionals

Four participants voluntarily reported having changed health professionals due to dissatisfaction with quality of care, a finding in accord with previous research [11, 18]. Interestingly, each of these participants also reported satisfaction with the care provided by their subsequent health professional.

#### 3.3.4. Psychosocial Factors

With respect to patients' fears and concerns, 29 (45.3%) participants reported that they received inadequate support. Concern was highest in relation to cancer recurrence and the impact on their children's health. Having a coordinator (defined as a person to assist in organising follow-up care) was valued by 40 (62.5%) participants. The qualitative analysis supports this finding with many participants equating coordination with improved support and information provision. Whilst some participants felt that they themselves did not require a coordinator, for a few participants this was constructed as entirely due to personal characteristics: these participants reported that they were organised and were confident. Strong support for a care coordinator in concert with concerns about gaps in care and communication (Box 1) indicates that, for these participants, structural support for melanoma follow-up care within the health care system was inadequate. In addition, participants indicated (Box 1) that lack of consistent support and information compounded the stress and fear associated with melanoma follow-up. Moreover, provision of reassurance from health professionals was constructed as an essential element of quality of care.

## 4. Discussion

### 4.1. Patient Satisfaction with Quality of Care

Unlike previous research into patient perceptions of the quality of melanoma follow-up care in Australia [9], our findings indicate considerable dissatisfaction with the quality of care received. The sampling of patients via websites and online support groups provides a cohort of melanoma patients including more nonmetropolitan participants and participants from multiple states than those reported in Morton et al., where participants were recruited from a specialist follow-up centre [9].

The findings suggest that there was considerable variability in perceived quality of care, and for many participants in our study, psychosocial support and information provision were inadequate. Both factors appeared to affect patients' sense of control, a finding mirrored in previous research [13].

### 4.2. Technical Aspects of Care

This study also identified considerable variance in follow-up practice in our participants' group with variation in the types of doctors seen and frequency of visits and indications of duplication of care. This finding mirrors a 2011 Netherlands study, where patients were reportedly receiving more follow-up visits than being clinically indicated [7]. In addition, Australian and New Zealand guidelines recommend that patients themselves should play a central role in monitoring for recurrence or new primary melanomas. Our findings suggest that essential education for self-examination, to support such monitoring, may be lacking.

The Australian and New Zealand guidelines do not recommend radiological tests (CXR, CT, and PET) in early stage melanoma [6] with the evidence indicating that routine imaging has minimal value in follow-up, and the additional cost cannot be justified [6, 19]. Some participants reported they would like more testing, and others reported excessive testing across the spectrum of disease. However, our findings must be interpreted with caution, as assessing the “appropriateness” of imaging for each patient (e.g., based on patient, tumour, and treatment characteristics) was beyond the scope of this study.

### 4.3. Interpersonal Aspects of Care

Our findings indicate that adequate provision of information was lacking for many patients. Lack of information and poor communication were associated with seeking information online, but this did not always provide an adequate alternative. High internet usage for unmet needs has been reported elsewhere [20] and attests to the desire of patients to be fully informed.

Participants reporting they had forgotten information used the terms “explosion” and “shock” (Box 1), suggesting intense emotional reaction to diagnosis of melanoma, may make it difficult to retain information provided. Patients may not recall information received during this time. Previous studies have indicated the need for ongoing tailored discussions with patients about their care throughout the “cancer journey” [21, 22].

Patient fears for their children's health and their own cancer recurring are reasonable given the evidence of recurrence risk and familial predisposition [6, 23]. Despite evidence that such concerns may be decreased with education and support [23, 24], our study suggests that many patients with melanoma receive inadequate support for psychosocial issues. Oliveria et al. showed that patient perceptions of inadequate support through the health care system could lead to suboptimal health outcomes [25]. Provision of a care coordinator, expressed as being of value by over half of the study participants, has been demonstrated to improve health outcomes in breast cancer patients [26] and may go some way to providing mental and emotional support.

## 5. Limitations

The online nature of the study presents selection bias and although potentially widely accessible across Australia, the number of participants was small. Therefore, the findings may not be representative of national experience of patients undergoing melanoma follow-up. As a retrospective study, the possibility of recall bias is also acknowledged. Reliance on categorical data limited analysis, making it difficult to assess relationships amongst variables.

## 6. Conclusion

This study provides insights into the nature of melanoma follow-up care in Australia. We identify perceived gaps in patient care, perceptions of inadequate support and information, and variance in patterns of care, all of which suggest that the quality and consistency of melanoma follow-up care in Australia can be improved. Moreover, follow-up care is iterative and the needs of patients, including information, emotional support, and medical care, may be very different from their needs during the treatment phase. According to our data, it appears that these changing needs are not being widely addressed. Moreover, the variation in patterns of care suggests the Australian and New Zealand clinical practice guidelines are not being consistently followed. Our data suggest that provision of targeted support and information from health professionals may improve long-term patient self-care. Whilst this finding deserves greater attention in future research, we also suggest that a focus on developing strategies for generating greater adherence to the clinical guidelines, including the stringent use of investigations as recommended, may generate a cost dividend which could be reinvested in preventive and supportive care. We suggest that greater consistency in the provision of emotional support and information throughout treatment and follow-up phases of melanoma follow-up care could enhance patient well-being.

## Supplementary Material

The 40-question survey, predominantly with “tick box” format, collected information about patient demographics, nature of the melanoma, treatment details and nature of follow-up care.

## Figures and Tables

**Box 1:**
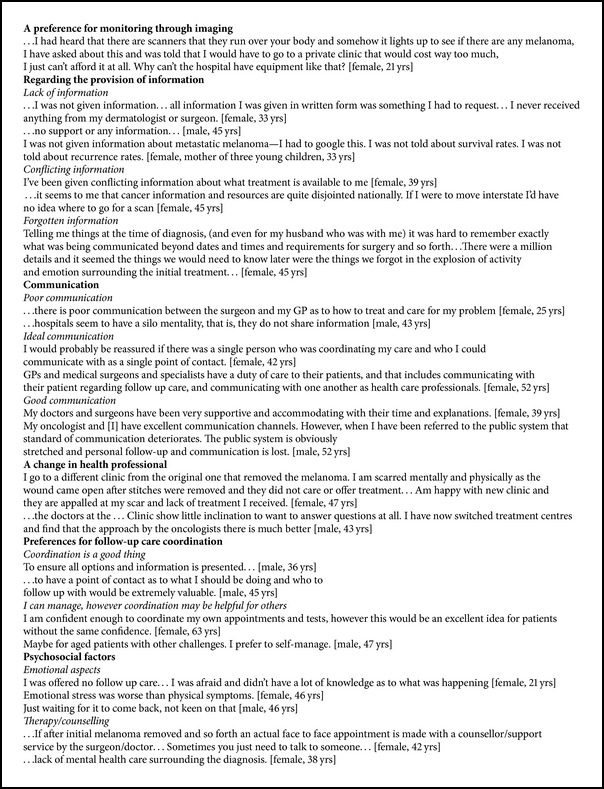
Examples of participant comments.

**Table 1:** Characteristics of patients (*n* = 64).

Characteristics		Number of patients
Median age in years (range)	50 (21–78)	64

Sex	Male	25
	Female	39

Highest education level	Year 11 or lower	10
	Year 12/HSC	6
	Certificate/trade/apprenticeship	9
	Diploma	11
	Degree or higher	27
	(missing)	1

Employment status	Full-time employee	33
	Part-time employee	14
	Unemployed	1
	Home duties	4
	Retired	12

Breslow thickness	<1 mm	13
	1–3 mm	23
	>3 mm	15
	Not sure	11
	(missing)	2

Lymph nodes involved	Yes, at time of diagnosis	7
	Yes, at a later time	14
	No	34
	Not sure	5
	(missing)	4

Patients with metastatic disease	Yes	7
	No	54
	Not sure	3

Treatment	Completed	40
	Ongoing	15
	Unsure	8

Secondary disease	Secondary melanoma	9
	Secondary nonmelanoma	18

Follow-up physician	General practitioner	31
	Dermatologist	29
	Skin clinic	13
	Surgeon	32
	Oncologist	12
	Radiotherapist	5
	Other	2

BRaf	Yes	7
	No	50
