# Supplementary material for: The Experience of Melanoma Follow-Up Care: An Online Survey of Patients in Australia
Source: J Skin Cancer. 2014 Nov 19;2014:429149. doi: 10.1155/2014/429149 (PMC4254069; doi:10.1155/2014/429149)
Supplement: Supplementary file 1 — The 40-question survey, predominantly with “tick box” format, collected information about patient demographics, nature of the melanoma, treatment details and nature of follow-up care. [file 429149.f1.pdf]

Invitation to Participate

This research is being conducted with the aim of understanding the experience of melanoma patients at their follow-up consults after the completion of their treatment. If you were diagnosed with a melanoma in Australia from 1 January 2007, and it is more than one year since your treatment, you are invited to participate.

The study is being undertaken as a Master of Public Health project at The University of Adelaide. It will be conducted under the supervision of experienced researchers at The University of Adelaide and a surgeon experienced with melanoma care. Supporting organisations and associated Patient Networks concerned with melanoma care are assisting the study by making the project available through their websites.

YOU WILL NEED TO READ THE PARTICIPANT INFORMATION SHEET AND CONSENT FORM, AND THEN GIVE YOUR CONSENT TO PARTICIPATING IN THE RESEARCH BEFORE YOU CAN UNDERTAKE THE QUESTIONNAIRE.

Yours sincerely,
Janine Mitchell
Research Student

Participant Information Sheet

Dear Participant,

Research projects conducted for medical issues can provide valuable information that may be used to improve patient health care and health outcomes. Please read this information sheet that will give an overview of this project and your role if you consent to participate.

PURPOSE OF STUDY
Patients who have finished their treatment for a melanoma then start follow-up care (usually about one year after their melanoma diagnosis). Effective follow-up care for patients who have had a melanoma is essential to monitor for recurring or new melanoma, to provide education, support, full body skin examinations and related tests.

The purpose of this research is to investigate and understand the experience and needs of melanoma patients in Australia at their follow-up consults. At the present time there is very little understanding of this experience from the melanoma patients' perspective. Identifying the current practice in melanoma follow-up care in Australia may provide data leading to improvements in this care. Although the study results may not directly benefit the survey participants, data collected may benefit their families and melanoma patients throughout Australia and internationally.

RESEARCH TEAM
Approval to conduct this project has been obtained from The University of Adelaide Human Research Ethics Committee. The Research Team members are as follows:

- Dr Jackie Street PhD, BSc (Hons), Grad.Dip.PHC ---- Ph: 08 8313 6498
- Janine Mitchell RN BNP Grad Dip (Masters student) PH ---- Ph: 0412 180 757
- Assoc/Prof Susan Neuhaus CSC, MBBS, PhD, FRACS ---- Ph: 08 8213 1800
- Dr Taryn Bessen MBBS FRANZCR ---- Ph: 08 8313 0603

PROJECT TITLE
What are the patients told, who do they see and what tests are they offered in melanoma follow-up? : An online survey of melanoma patients in Australia.

SURVEY QUESTIONNAIRES
You are invited to participate in an online questionnaire that is expected to take about 5-10 minutes to complete. Questions will be set out in four sections, predominantly in an easy 'tick box' format. The first section will ask for some details such as your age and the state your melanoma was treated in. The following two will cover details of your treatment and then follow-up care. The final section will have a few closing questions. You are not required to provide your name or any other identifying details.

We would like to reassure you that your relationship with the supporting associations will not be affected in any way if you do not wish to participate and you may withdraw from the study at any time without prejudice.

We understand that there is a possibility that you may become upset when describing your personal experience with melanoma. If this should occur, please discontinue the questionnaire and if you wish contact a trusted health professional or your GP. Alternatively you may wish to contact the Cancer Council Ph: 131120. This is a free, confidential telephone information and support service (9am-5pm, Mon-Fri) run by Cancer Councils in each state and territory and is a local call cost from anywhere in Australia.

[Please continue to the next page for further information]

Participant Information Sheet (continued)

STORAGE OF QUESTIONNAIRES
Data collected from the questionnaires will be entered into a secure computer data processing package in order to analyse the information. Your questionnaire responses will be kept confidential and only the collected unidentified data will be used in reports or publications from the study. Data from the questionnaires will only be viewed by the research team and no other party.

If you wish to receive a copy of the project results you will need to provide your name and contact details at the end of the survey. In this case, the questionnaire and name will be immediately separated when returned after completion and the information stored separately in two secure sites to ensure confidentiality and your anonymity. This information will not be shared with any other party.

PROJECT RESULTS
The project data and results will be used towards Janine Mitchell's Masters degree and will be published in a journal article, with no identifying participant data. A summary will be sent to participants if requested. Associations affiliated with this project will receive a project summary. This project is the first in a proposed series of studies and there will be an opportunity to indicate if you are willing to be contacted to participate in follow-on research.

As per protocol, The University of Adelaide Complaints Form is included in this information.

CONTACTS FOR INFORMATION ON PROJECT AND INDEPENDENT COMPLAINTS PROCEDURE

The following study has been reviewed and approved by The University of Adelaide Human Research Ethics Committee:

Project Title: What are the patients told, who do they see and what tests are they offered in melanoma follow-up ? : An online survey of melanoma patients in Australia.
Approval Number: H-2012-037. This number is given once the project has been approved.

The Human Research Ethics Committee monitors all the research projects which it has approved. The committee considers it important that people participating in approved projects have an independent and confidential reporting mechanism which they can use if they have any worries or complaints about that research. This research project will be conducted according to the NHMRC National Statement on Ethical Conduct in Human Research (see http://www.nhmrc.gov.au/publications/synopses/e72syn.htm)

1. If you have questions or problems associated with the practical aspects of your participation in the project, or wish to raise a concern or complaint about the project, then you should consult the project co-ordinator:
Dr Jackie Street PhD, BSc(Hons), Grad.Dip.PHC
Ph: +618 8313 6498
Email: Jackie.Street@adelaide.edu.au

2. If you wish to discuss with an independent person matters related to:
- making a complaint, or
- raising concerns on the conduct of the project, or
- the University policy on research involving human participants, or
- your rights as a participant,

contact the Human Research Ethics Committee’s Secretariat on phone (08) 8303 6028.

Please contact a member of the Research Team if you have any questions about this project.
COMPLETED QUESTIONNAIRES WILL BE ACCEPTED UP UNTIL 30 SEPTEMBER 2012.

Yours sincerely,
Janine Mitchell (on behalf of the Research Team)

Consent Form

I consent to take part in the research project titled: What are the patients told, who do they see and what tests are they offered in melanoma follow-up? : An online survey of melanoma patients in Australia.

I acknowledge I have been provided with the following information from Janine Mitchell on behalf of the Research Team:

1. This research is being undertaken as part of a Master's of Public Health degree at The University of Adelaide
2. I have been informed of the nature, purpose and possible effects of the research
3. My relationship with the supporting association will not be affected if I choose not to participate
4. My involvement in this research project may not be of any direct benefit to me
5. I am free to withdraw from the project at any time without prejudice
6. The questionnaire will take about 5-10 minutes to complete
7. The results of this study will be written as an article, be published in a journal and may be presented at conferences, but the information will be provided in such a way that I cannot be identified.

• My consent is given voluntarily.

• I have understood and am satisfied with the explanations that I have been given.

• I have been provided with a written information sheet.

• I declare that I am over the age of 18 years.

Please complete the statement below.

***1. I have read, understood and acknowledge the above and give my consent to participate.**

○ Yes ○ No

PATIENT DETAILS

The following questions will help us to determine your background and provide us with details of your personal experience of having a melanoma. Questions marked with an asterisk* require an answer as these are essential for some of the project results.

Please note that there is an opportunity for you to make any additional comments at the end of the survey.

*2. Is it more than 12 months since you were diagnosed with a melanoma?

- ○ Yes
- ○ No

*3. Age at last birthday

*4. Sex

- Male
- Female
- Transsexual/Transgender

5. What is the highest level of education you have completed?

- Year 11 or lower
- Year 12/Higher School Certificate
- Certificate/Trade/Apprenticeship
- Diploma
- Degree or higher

6. What best describes your employment situation?

- Employed full time
- Employed part time
- Unemployed
- Home duties
- Retired

7. What is your history of sun exposure? How many times did this occur? (tick all that apply)

	Never	Rarely	Occasionally	Often
Sunburnt as a child (up to age 18)				
Sunburnt as an adult				
Sun bathing				
Outdoor sport				
Working outdoors				
Solarium				

8. How would you describe your skin type? (tick all that apply)

- ☐ Very fair/light - burns easily
- ☐ Light skin tone - will tan a little
- ☐ Light to olive - usually tans
- ☐ Medium brown
- ☐ Dark brown to black
- ☐ Numerous moles

9. Have any of your blood relatives (grandparents, parents, siblings, children) been diagnosed with a melanoma?

- ○ Yes
- ○ No
- ○ Not sure

10. Have any of your blood relatives been OFFERED a full body skin assessment to check for a melanoma?

- ○ Yes
- ○ No
- ○ Not sure

11. Have any of your blood relatives RECEIVED a full body skin assessment to check for a melanoma?

- ○ Yes
- ○ No
- ○ Not sure

12. When were you diagnosed with a melanoma (approximately if not sure)?

- ○ Before 2007
- ○ 2007
- ○ 2008
- ○ 2009
- ○ 2010
- ○ 2011
- ○ Not sure

13. In which of the following was your treatment and follow-up care?

- ☐ SA
- ☐ VIC
- ☐ ACT
- ☐ NSW
- ☐ QLD
- ☐ NT
- ☐ WA
- ☐ TAS

TREATMENT OF YOUR MELANOMA

14. Do you know how thick (BRESLOW thickness) your melanoma was at diagnosis ? If known, please select thickness below.

- <1mm
- 1-3mm
- >3mm
- Not sure

15. Have you been given a copy of the pathology report?

- Yes
- No
- Not sure

16. Were your lymph nodes involved?

- Yes, at time of diagnosis
- Yes, at a later time
- No
- Not sure

17. Have you had metastatic disease (eg cancer spread to lung, liver, bowel, bone or brain)?

- Yes
- No
- Not sure

18. Have you completed your treatment?

- Yes
- No
- Palliative care
- Not sure

19. Who did you see for your melanoma treatment? (tick all that apply)

	Public	Private	Not sure
General Practitioner	☐	☐	☐
Dermatologist	☐	☐	☐
Skin clinic (eg Molescan)	☐	☐	☐
General/Plastic/Cancer Surgeon	☐	☐	☐
Oncologist	☐	☐	☐
Radiotherapist	☐	☐	☐
Not sure	☐	☐	☐

20. What treatment have you received? (tick all that apply)

- ☐ Surgery to remove the skin melanoma
- ☐ Sentinel lymph node biopsy
- ☐ Lymph node dissection
- ☐ Surgery to remove melanoma spread inside the body (eg lung, liver, bowel, bone, brain)
- ☐ Chemotherapy
- ☐ Immunotherapy (eg Interferon)
- ☐ Radiotherapy
- ☐ Participant in a clinical trial

21. Have you developed a second skin cancer since the completed treatment of your first melanoma?

	Yes	No
Melanoma	○	○
Non-melanoma	○	○
Not sure	○	○

FOLLOW-UP CARE

PLEASE READ THE FOLLOWING:
Patients who have completed their treatment for a melanoma then start FOLLOW-UP CARE (usually about one year after diagnosis). This may include regularly scheduled visits to your skin doctor for checks such as a full body skin assessment and possible tests.

22. What type of health professionals have you seen for your follow-up care? Please tick which you have seen and how often you have seen them.

	3 month	6 month	12 month	More than 12 months
General Practitioner	○	○	○	○
Dermatologist	○	○	○	○
Skin clinic (eg Molescan)	○	○	○	○
General/Plastic/Cancer Surgeon	○	○	○	○
Oncologist	○	○	○	○
Radiotherapist	○	○	○	○
Other	○	○	○	○

If 'Other', please list

23. Which of the following tests were you OFFERED at FOLLOW-UP? Who offered them?

	B-RAF mutation test	Chest x-ray	CT Scan	PET Scan
General Practitioner	☐	☐	☐	☐
Dermatologist	☐	☐	☐	☐
Skin clinic (eg Molescan)	☐	☐	☐	☐
General/Plastic/Cancer Surgeon	☐	☐	☐	☐
Oncologist	☐	☐	☐	☐
Radiotherapist	☐	☐	☐	☐
Not sure	☐	☐	☐	☐

24. How many of each of the following tests have you RECEIVED during FOLLOW-UP?

	None	1	2-5	6-9	10 or more	Not sure
Chest X-ray	○	○	○	○	○	○
CT Scan	○	○	○	○	○	○
PET Scan	○	○	○	○	○	○

25. Have you ever RECEIVED a B-RAF mutation test to check for melanoma risk?

○ Yes

○ No

○ Not sure

26. Have any of your family been OFFERED a B-RAF mutation test for melanoma risk?

- Yes
- No
- Not sure

27. Do you have a WRITTEN melanoma follow-up plan?

- Yes
- No

28. Would it be of value for patients having melanoma follow-up care to have a Coordinator: somone who would arrange appointments and tests, and ensure good communication between all the health professionals?

- Yes
- No
- Not sure

Please feel free to comment.

29. Please select the answer that best describes how much you worry about each of the following.

	Not at all	A little bit	Somewhat	Very much
Future diagnostic tests	○	○	○	○
Another type of cancer	○	○	○	○
My cancer coming back	○	○	○	○
Dying	○	○	○	○
My health	○	○	○	○
My children's health	○	○	○	○

30. In terms of your fears and concerns about your melanoma diagnosis and treatment, do you feel there has been adequate support for you during your follow-up care?

- Yes
- No

If you answered No, please briefly explain your response.

31. During your follow-up visits, have you received WRITTEN information on the following?

	Yes	No
Melanoma	○	○
Cancer services	○	○
Patient support websites	○	○
SUNSMART advice (slip, slop,slap etc)	○	○
To avoid Solariums	○	○

32. Thinking about the amount of information you were given, would you have liked:

- ○ Less information
- ○ More information
- ○ The same amount of information

33. Have you used any of the following services?

	Yes	No
Cancer services	○	○
Patient support websites	○	○

Please list (if known)

34. What has been your MAIN source of information on melanomas?

- ○ General Practitioner
- ○ Surgeon
- ○ Dermatologist/Skin clinic (eg Molescan)
- ○ Books/brochures
- ○ Websites
- ○ Family/friends
- ○ Other

If 'Other', please specify

35. When did you receive this information on melanomas?

- ○ Before diagnosis
- ○ During treatment
- ○ During follow-up care

36. Is there anything else important about follow-up care that you think we should know about?

- Yes
- No

Please write your comments here

37. Were there any questions in this survey that you did not understand or had trouble answering?

- Yes
- No

If yes, please explain which questions and why

CLOSING COMMENTS

WE WOULD LIKE TO THANK YOU FOR YOUR TIME AND CONTIBUTION IN COMPLETING THIS QUESTIONNAIRE.
IF YOU WOULD LIKE TO RECEIVE THE FINDINGS OF THIS SURVEY OR WOULD BE WILLING TO PARTICIPATE IN FURTHER RESEARCH PLEASE INDICATE BELOW.

38. Would you like to receive a summary of the results of this project?

- Yes
- No

39. Would you be willing to be contacted to participate in similar research in the future?

- Yes
- No

40. If you answered YES to Q38 or Q39 please provide your name and email address below. [Note: these details will be stored separately to your survey answers]
